# Autologous blood resuscitation for large animals in a research setting using the Hemafuse device: Preliminary data of device use for controlled and real-world hemorrhage

**DOI:** 10.3389/fvets.2022.1069420

**Published:** 2022-12-22

**Authors:** Rebecca N. Treffalls, Matthew Lubas, Jonathan J. Morrison, David P. Stonko

**Affiliations:** ^1^R. Adams Cowley Shock Trauma Center, University of Maryland, Baltimore, MD, United States; ^2^Sisu Global Health, Baltimore, MD, United States; ^3^Department of Vascular and Endovascular Surgery, Mayo Clinic, Rochester, MN, United States; ^4^Department of Surgery, Johns Hopkins Hospital, Baltimore, MD, United States

**Keywords:** resuscitation, hemorrhage, autologous blood, Hemafuse, large animals, whole blood, blood bank

## Abstract

**Introduction:**

New low-cost technologies are needed to salvage lost blood in low-resource settings and large animal laboratories. The Hemafuse device is a simple mechanical device that can recover lost blood during surgery. The aim of this study is to assess the feasibility of this device for resuscitating large animals with controlled and unintended hemorrhage and to provide device considerations for use in this context.

**Methods:**

This study had two experimental components: (1) the Hemafuse device was kept on-shelf and used as needed to assess real-world use for unintended hemorrhage during experiments, and (2) animals underwent a controlled hemorrhage protocol, where four anesthetized swine underwent aortic and external jugular vein catheterization for pressure monitoring. Animals were hemorrhaged into the pelvis, and the Hemafuse device was used to suction the blood through a filter and pushed into a heparinized bag for subsequent retransfusion. Blood samples were collected at baseline, hemorrhage, within the device, and post-retransfusion and laboratory tests were performed.

**Results:**

Animals that underwent controlled hemorrhage had a baseline mean arterial pressure of 83.6 ± 7.8 mmHg, and central venous pressure of 12.8 ± 1.9 mmHg, with expected changes throughout hemorrhage and resuscitation. Following resuscitation, pH was similar to baseline (7.39 ± 0.05 vs. 7.31 ± 0.03, *p* = 0.24). Lactate increased throughout the experiment with no significant differences after autotransfusion compared to baseline (2.7 ± 0.7 vs. 4.1 ± 1.4 mmol/L, *p* = 0.37). There were no significant changes in metabolic physiology. Compared to baseline, the hemoglobin (7.8 ± 2.4 vs. 7.3 ± 1.8 g/dL, *p* = 0.74), hematocrit (23% ± 6.9 vs. 21.3% ± 5.6, *p* = 0.71), and activated clotting time (268.5 ± 44.5 vs. 193 ± 24.6 s, *p* = 0.35) were similar after retransfusion. When used for unintended hemorrhage, the animals were resuscitated using the device with a mean time to retransfusion time of 128.7 ± 13.3 s and 100% survival throughout the experiment.

**Conclusion:**

The Hemafuse device is feasible and efficacious for supporting large animal resuscitation. This is preliminary evidence that the device is a low-risk and low-cost off-the-shelf option for resuscitation using autologous blood with no significant effect on physiology post-retransfusion. We recommend that research laboratories consider the Hemafuse device for emergency use, particularly for highly invasive surgical laboratories where banked blood is not readily available.

## Introduction

Many large animal laboratories utilize invasive techniques, such as surgical or endovascular methods, to design, assess, validate, and innovate across the breadth of the biomedical field (1–5). Unfortunately, during these developmental stages and in what are often low-resource animal labs, attrition is not uncommon, with studies often calculating sample size based on an expected 10% attrition rate (6, 7). One cause of reversible attrition is unintended hemorrhage, which can be reduced by preventing hemorrhage in the first place, or prompt resuscitation (8, 9). In humans, optimal resuscitation strategies for non-compressible hemorrhage, where the mortality rate is between 20 and 30%, have been extensively studied with emphasis on early blood transfusions (9–11). While blood transfusions are effective for resuscitating humans; some large animal laboratories may have limited resources without access to banked animal blood—especially in experiments where transfusion is not anticipated. Crystalloids are easily obtained, making fluid resuscitation a feasible option, but this lacks the efficacy of blood products and mainly serves as an adjunct therapy rather than a sole resuscitative method in large-volume hemorrhage (12, 13).

Resuscitation following unintended hemorrhage in a large animal laboratory is challenging. Transfusion using the autologous blood recovered during hemorrhage presents a potential method of resuscitation for large animals (14). Current methods of autotransfusion, such as the intraoperative cell saver (ICS) machine, are effective but can have inherent limitations, such as cost, maintenance, and need for specialized training (i.e., a perfusionist) (14–18). In translational research laboratories, maintaining normal physiology while performing surgical experiments can be paramount to drawing scientific conclusions. Whereas, in a clinical setting, the primary outcomes of surgery are generally patient-centered. In some instances, animal experiments may be terminated due to physiologic derangement, even if the animal is recovered. Quickly resuscitating a large animal to complete an experiment can preserve precious resources and lessen the cost burden of attrition by reducing the total number of animals required to meet the planned sample size. Overall, new low-cost technologies are needed that can be used with little training to salvage lost blood from large animals for retransfusion of autologous blood.

Recently, a simple, mechanical method of autotransfusion, the Hemafuse device, was engineered by Sisu Global Health for low-resource facilities to quickly return shed blood back to a human patient or animal undergoing surgery (19). The pooled blood is suctioned through a yankauer suction tip, filtered through a handheld device, pushed into a pre-anticoagulated bag, and re-transfused to the patient. This low-cost method of autotransfusion could prove beneficial in large animal laboratories to provide blood transfusion to animals and to salvage animals after blood loss. We sought to determine the feasibility and efficacy of the Hemafuse device for resuscitation in a large animal using two methods: real-world use for resuscitation following unintended hemorrhage and resuscitative use in a model of controlled large volume open intraperitoneal pelvic hemorrhage. In addition to device feasibility, this report includes device considerations and technical troubleshooting for use in large animals.

## Materials and methods

### Animal selection

All procedures performed were approved by the University of Maryland School of Medicine institutional animal care and use committee under various protocols. All protocols utilized castrated male Yorkshire swine weighing between 50 and 70 kg. The animals were housed in individual cages next to each other with free access to food and water under the supervision of licensed veterinary staff. Animals were fasted for 12 h prior to the operation. A similar experimental protocol regarding Hemafuse device use for controlled intraperitoneal hemorrhage in swine has been described in detail by Treffalls et al. (20).

### Animal preparation and monitoring

All animals followed our usual sedation, anesthesia, and monitoring protocols. They were sedated with midazolam (0.2–0.5 mg/kg) followed by telazol (4–5 mg/kg) and xylazine (1.8–2.2 mg/kg) *via* intramuscular injection. Animals were transported to the laboratory with 100% oxygen and induced with inhaled isoflurane (3–5%) *via* a facemask. Following endotracheal intubation, sedation was maintained under inhalational isoflurane (1–3%) and titrated to a minimum alveolar concentration (MAC) of 1.0–1.4. Mechanical ventilator settings were placed on volume-controlled mode with FiO2 at 40%, tidal volume set to 8–12 mL/kg, and a respiratory rate titrated to maintain an end-tidal CO2 between 30 and 40 mmHg and maintained with arterial blood gas (ABL800 FLEX blood gas analyzer; Radiometer, Brea, CA).

The minimum physiologic monitoring set up in our laboratory consists of percutaneous arterial access with a solid-state pressure probe (Transonic Corporation, Ithaca, NY) inserted for mean arterial pressure (MAP), percutaneous venous access with a pressure probe inserted for central venous pressure (CVP), electrocardiography for heart rate and electrical activity, and rectal temperature probe. All physiologic data is continuously acquired using the AD instruments PowerLab and LabChart data collection system (LabChart and Powerlab, ADInstruments, Sydney, Australia).

### The Hemafuse device

**Figure 1:**
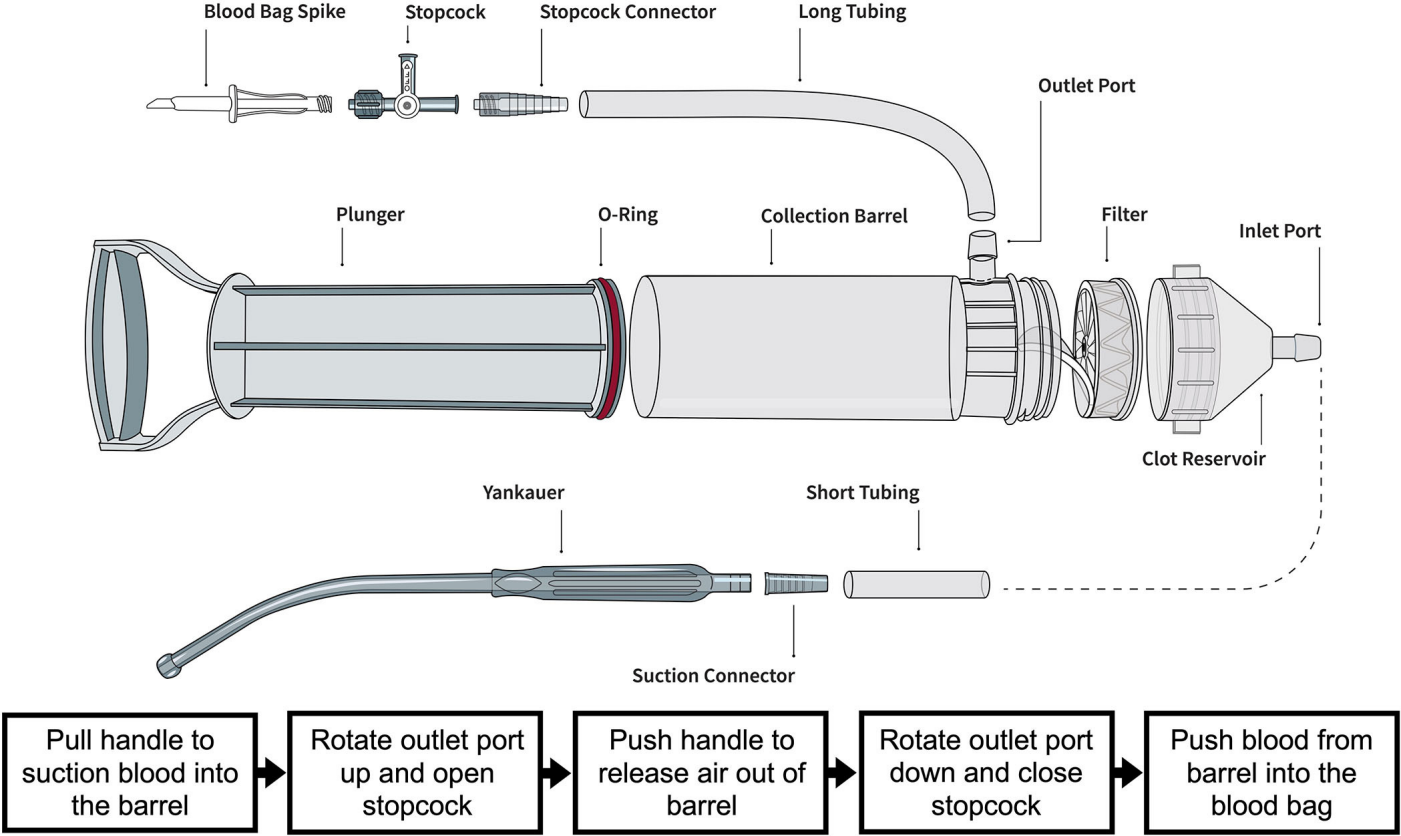
Illustration of the Hemafuse device and the individual parts courtesy of the Sisu Global (https://sisuglobal.health/brochures). Beneath the illustration is a step-by-step procedure on how to use the device: (1) Pull the handle to suction blood into the barrel, (2) ensure the outlet port up, and open the stopcock, (3) push the handle to release air out of the barrel, (4) rotate outlet port down, and close stopcock, and (5) push blood from the barrel into a heparinized blood bag.

**Figure 2:**
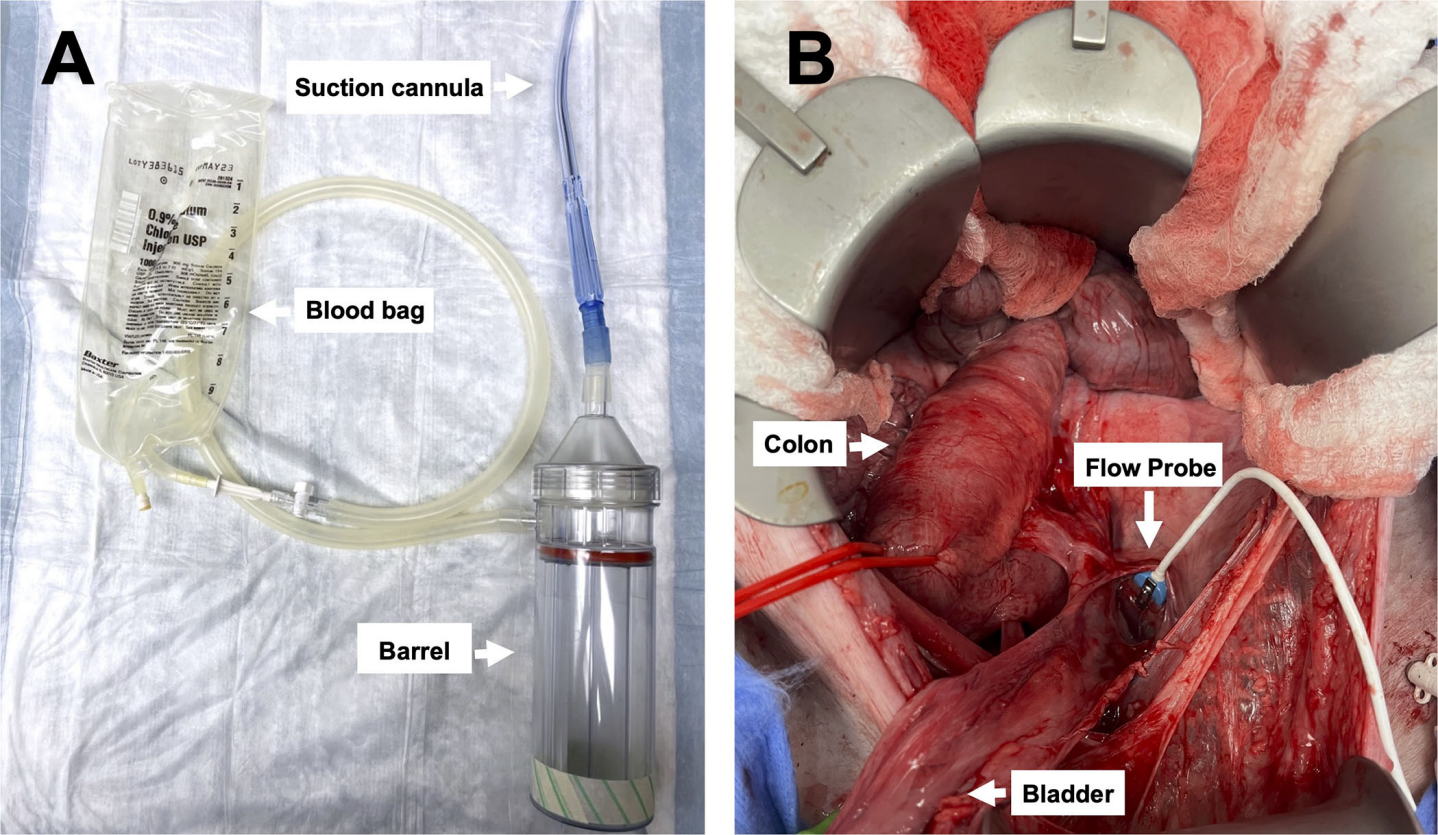
Images of the device and pelvic exposure: **(A)** The Hemafuse device (barrel) and the suction cannula are connected to an empty blood bag (i.e., an empty crystalloid 1L bag), and **(B)** surgical exposure of the pelvic well created for the pelvic hemorrhage model.

The Hemafuse device is made up of a pump, filter, and accessories kit (Figure 2A; Sisu Global Health, Baltimore, MD) that can be connected to any standard anti-coagulated blood bag (19). The filter is a 170-micron mesh filter that filters debris and particles and prevents blood clots from entering the barrel. This filter does, however, allow platelets to pass through the filter. In this study, the Hemafuse device was connected to a 1 L blood bag pre-heparinized with 10,000 Units. When the device is used, blood is suctioned into the barrel by pulling on the pump handle with the outlet port facing upwards. Following the completion of suction, air must be released out of the barrel by pushing the plunger with the outlet port facing up. After air priming and removal, the barrel is then rotated to have the outlet port facing down, and blood is pushed into the attached blood bag. The process repeats by collecting and filtering the pooled blood by pulling the handle and processing the blood from the pump into the blood bag by pushing the handle until all pooled blood is adequately suctioned or until the blood bag is full (Figure 1). If a blood bag is filled completely and there is remaining pooled blood, an additional blood bag can be connected during use. The blood and heparin within the blood bag were gently mixed by rocking the bag. The blood transfusion tubing was connected to the bag and retransfused into the jugular vein using gravity.

### Controlled hemorrhage model for device evaluation

To assess the use of this device for resuscitating large animals in a research setting, two adult male swine were enrolled. A model of endovascular pelvic hemorrhage in Swine has previously been established by Abdou et al. (21) which was adapted here into an open model of intraperitoneal pelvic hemorrhage. Following model development, we enrolled two adult male swine into a full experimental protocol.

#### Instrumentation

Animals were intubated as previously described, EKG leads were placed, and 6Fr sheaths (Terumo, Elkton, NJ) were percutaneously placed into the carotid artery and femoral vein for pressure monitoring. An 8.5Fr Cordis sheath (Cordis, Santa Clara, CA) was inserted into the external jugular vein for fluid administration and blood transfusions. An 8Fr sheath (Terumo, Elkton, NJ) was placed into the femoral artery and reserved for hemorrhage. A low midline laparotomy was performed for access to the pelvis. A cystotomy with foley catheter placement was performed for urine drainage. An iliac artery flow probe (6 mm; ADInstruments, Series MA-n-PS-ori) was placed on one experimental animal. The two experimental animals were left undisturbed for 30 min for baseline data collection of MAP, CVP, heart rate, and iliac artery flow.

#### Hemorrhage and resuscitation

Once instrumented, we developed a method to create a pelvic well-suited for hemorrhage. This was accomplished by providing retraction to the abdominal side wall with a Bookwalter retractor and cephalad retraction of the small and large intestines (see Figure 2B). Once a pelvic well was created, the femoral sheath opened into the pelvic well, and the animal was hemorrhaged until the cavity was full. The pelvic well-capacity was ~1 L of blood. Once the hemorrhage was halted, the Hemafuse device was used to suction the blood from the pelvis into the heparinized blood bag. The autologous blood was fully transfused into the animals. Intravenous crystalloid fluid was administered in the two experimental animals, and the animals were resuscitated for 90 min. Arterial blood gas and chemistry results were used to guide ventilation adjustments, whereas hematologic laboratory results and hemodynamics were used to guide resuscitation.

#### Laboratory monitoring

In the two controlled hemorrhage model development animals, arterial blood samples from the animal before hemorrhage, samples from the post-suction blood bag, and arterial blood samples from the animal post-transfusion were obtained. In the two controlled hemorrhage experimental animals, samples from the baseline arterial blood, post-suction blood bag, immediately post-retransfusion, and every 30-min during resuscitation were obtained. ABG, Chem8+, and activated clotting time (ACT) were analyzed in all animals.

### Unintended hemorrhage for real-world device evaluation

The device was left on the shelf for as-needed emergency use for 30 animals over three unrelated IACUC protocols. The device was reserved for resuscitative use in animals with unexpected hemorrhage. The time to re-transfusion was recorded, and laboratory tests, such as arterial blood gas (ABG) and Chemistry 8+, were taken post-transfusion.

### Statistical analysis

Statistical analysis was performed using Stata v17.0 (Stat Corp LLC, College Station, TX, USA), and graphical representation was performed using GraphPad Prism v8.0 (GraphPad Software Inc, San Diego, CA, USA). Descriptive data and univariate analysis were performed: (1) hemodynamic baseline data compared to retransfusion data and (2) baseline laboratory data compared to the blood bag and the retransfusion data.

## Results

### Controlled hemorrhage

Four male Swine (50–60 kg) were used for the model development (*n* = 2) and experimental protocol (*n* = 2). The surgical exposure was optimized, and an adequate pelvic well was developed (Figure 2B). The baseline MAP was 83.6 ± 7.8 mmHg, CVP was 14.7 ± 0.3 mmHg, and iliac artery flow was 201 ± 46 mL/min.

**Figure 3:**
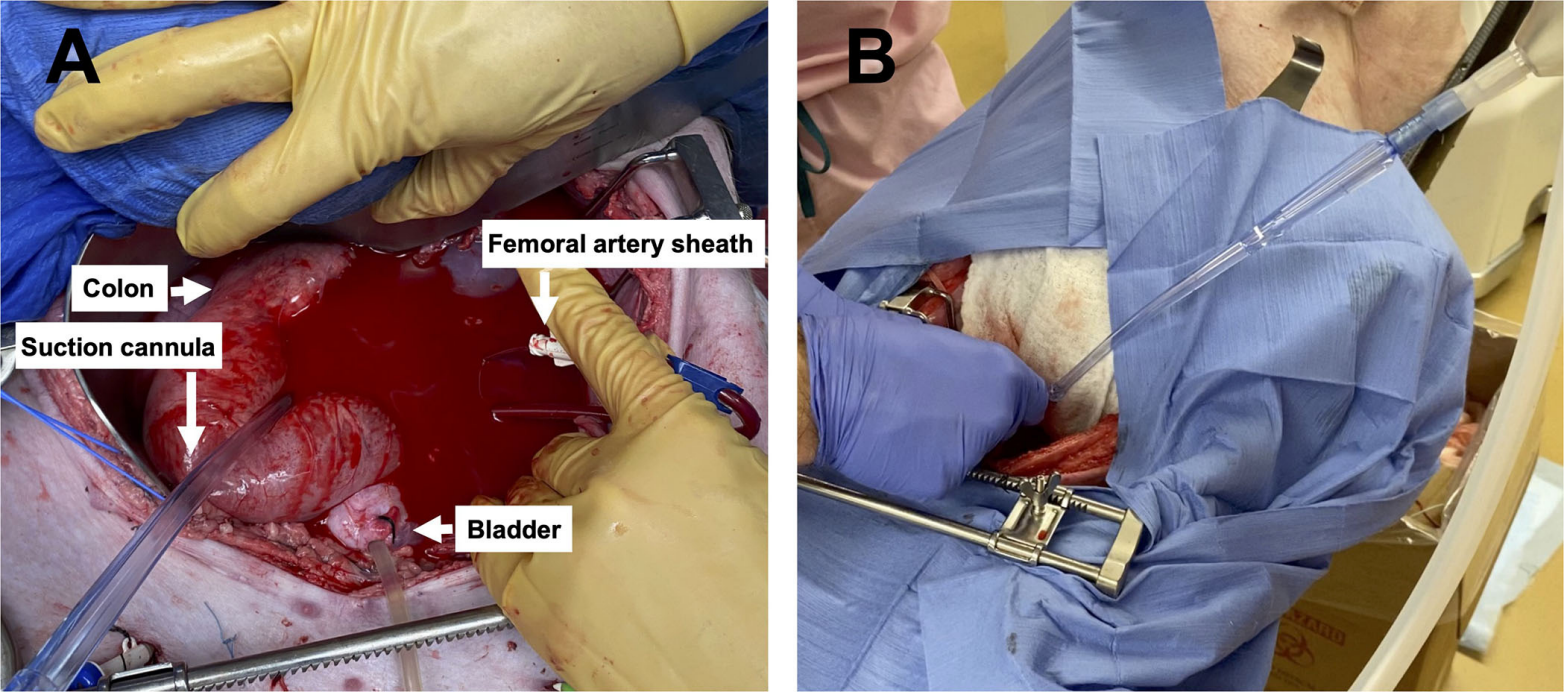
Images of the pelvic hemorrhage model and device set-up: **(A)** The pelvic exposure with the femoral artery sheath actively hemorrhaging into the pelvic well and **(B)** the set-up of the Hemafuse device with the suction cannula in the pelvic well.

**Figure 4:**
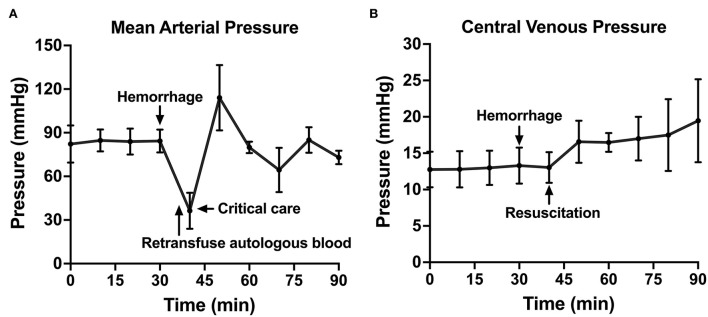
The hemodynamic variables across time: **(A)** Mean arterial pressure shown throughout baseline, hemorrhage, retransfusion of autologous blood, and a critical care period with intravenous fluid, if needed, and **(B)** central venous pressure shown throughout baseline, hemorrhage, and a similar resuscitation period.

**Figure 5:**
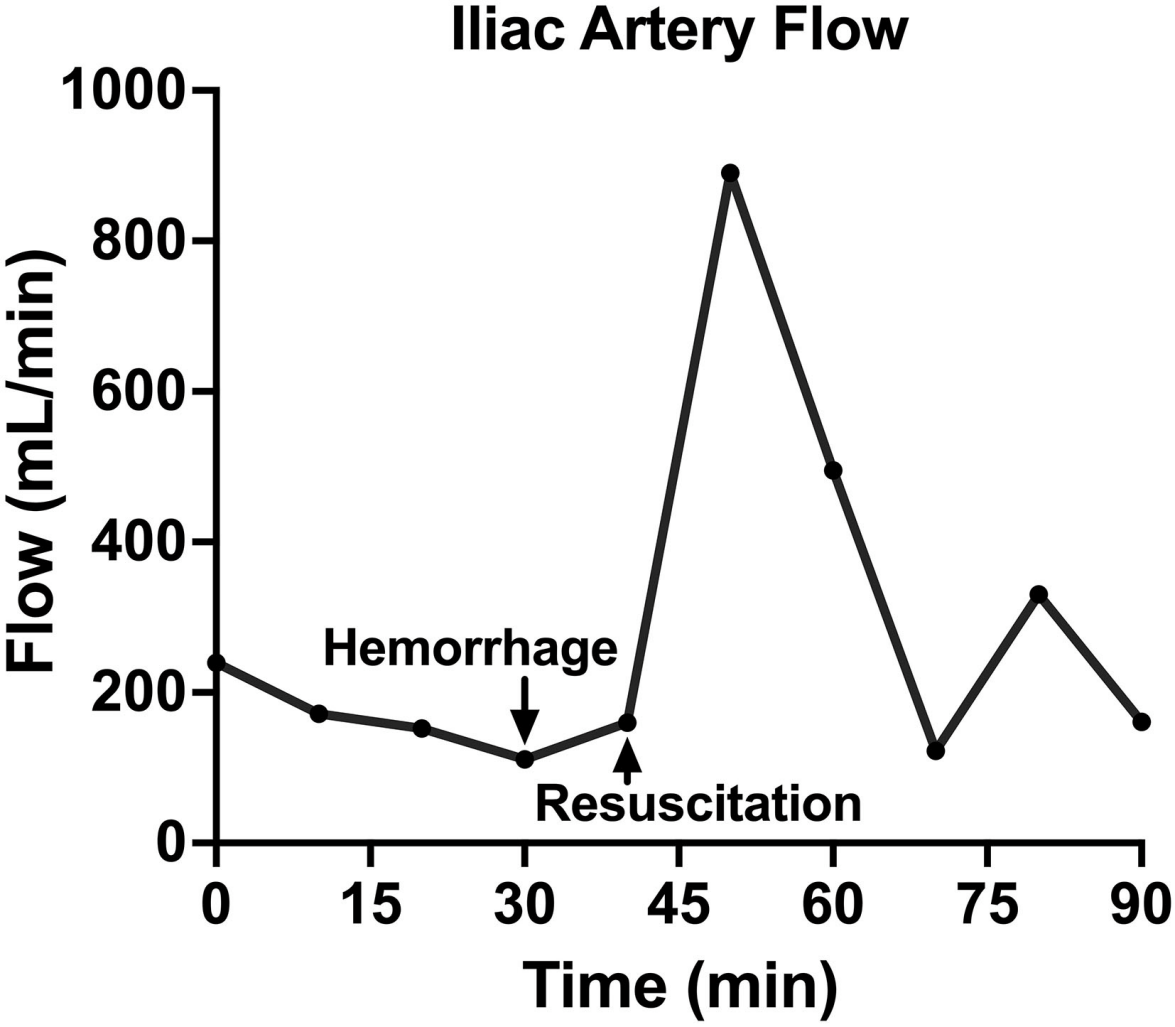
The iliac artery blood flow of one animal over time, including baseline, hemorrhage, retransfusion of autologous blood, and a critical care period.

Following instrumentation, the animal was hemorrhaged into the pelvic well (Figure 3A). The Hemafuse device was used to aspirate blood from the pelvis until the animal was bled approximately 20% of the animal weight (66 mL/kg) (Figure 3B). The MAP dropped to 36.3 ± 13.4 mmHg during hemorrhage and returned to baseline values during resuscitation (90.7 ± 21.8 mmHg, *p* = 0.48; Figure 4A). Compared to baseline, the CVP decreased during hemorrhage (11.9 ± 1.4 vs. 14.7 ± 0.3 mmHg; *p* = 0.01) and increased during resuscitation (17.6 ± 3.2 vs. 14.7 ± 0.3 mmHg, *p* = 0.02; Figure 4B). The iliac artery flow (*n* = 1) increased during resuscitation but normalized over time to baseline values (Figure 5).

**Figure 6:**
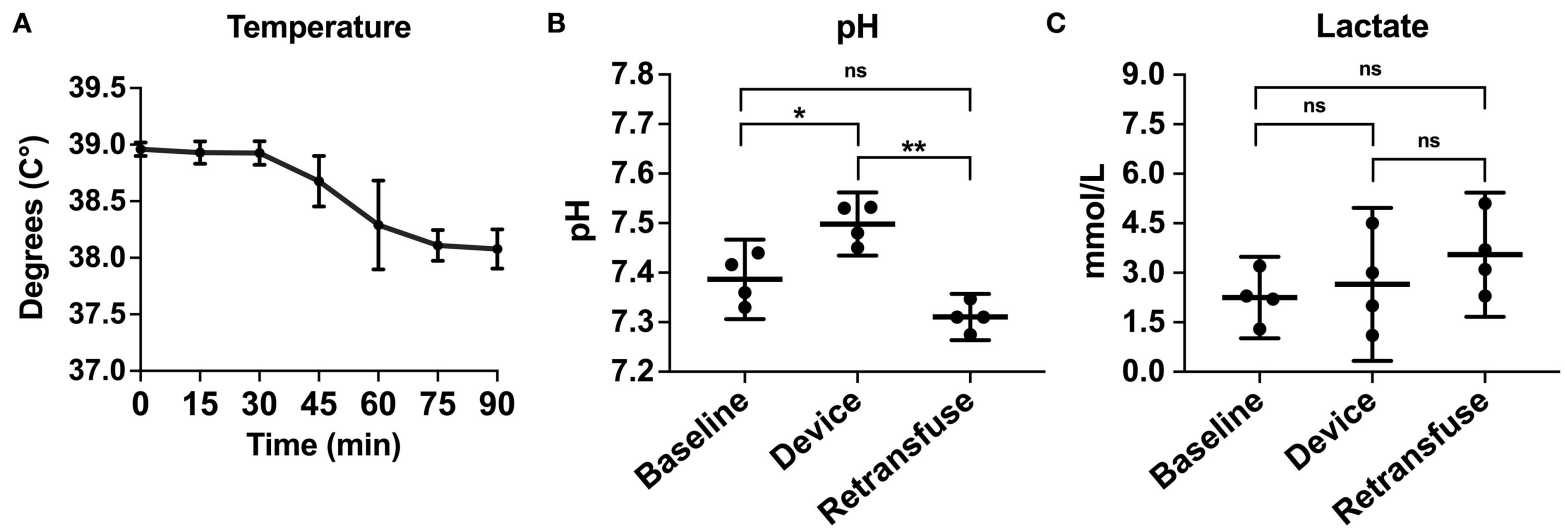
The metabolic laboratory tests were performed over time or between samples from the animal at baseline, within the device, and the animal at retransfusion: **(A)** Temperature of the animals across time. **(B)** pH at baseline, device, and retransfusion with significant differences between baseline vs. device and device vs. retransfusion but no difference between baseline vs retransfusion, and **(C)** lactate at baseline, device, and retransfusion without any significant differences. The central lines represent the means and the error bars represent the standard deviation. *p* < 0.05 is represented by *, a *p* < 0.001 is represented by **, and nonsignificant p values are presented by ns.

Compared to baseline data, temperature was not different during hemorrhage (38.9 ± 0.07 vs. 38.8 ± 0.20°C, *p* = 0.25) but was significantly lower during resuscitation (38.9 ± 0.07 vs. 38.2 ± 0.23°C, *p* < 0.001; Figure 6A). pH significantly decreased during hemorrhage (7.39 ± 0.05 vs. 7.28 ± 0.02, *p* = 0.02), however, during resuscitation, pH was similar to baseline (7.39 ± 0.05 vs. 7.31 ± 0.03, *p* = 0.24; Figure 6B). Lactate increased throughout the experiment, but there was no significant difference post-transfusion of autologous blood compared to baseline (2.7 ± 0.7 vs. 4.1 ± 1.4 mmol/L, *p* = 0.37; Figure 6C).

**Figure 7:**
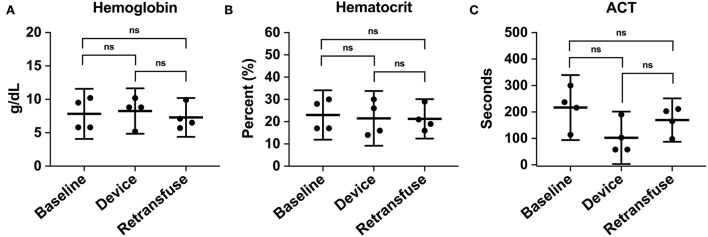
The hematologic laboratory tests were grouped into samples from the animal at baseline, within the device, and the animal at retransfusion: **(A)** Hemoglobin at baseline, device, and retransfusion without any significant differences. **(B)** hematocrit at baseline, device, and retransfusion without any significant differences, and **(C)** ACT at baseline, device, and retransfusion without any significant differences. The central lines represent the means and the error bars represent the standard deviation. Nonsignificant *p*-values are presented by ns.

A metabolic panel was similar to the retransfusion of autologous blood compared to baseline data (Table 1). There were no significant changes demonstrating hypocalcemia, hypokalemia, or hyperkalemia. Compared to baseline, the hemoglobin (7.8 ± 2.4 vs. 7.3 ± 1.8 g/dL, *p* = 0.74), hematocrit (23% ± 6.9 vs. 21.3% ± 5.6, *p* = 0.71), and ACT (268.5 ± 44.5 vs. 193 ± 24.6 s, *p* = 0.35) remained similar following retransfusion of blood (Figure 7).

**Table 1:** Metabolic, arterial blood gas, and hematologic laboratory data.

**Variable, Mean ±SD**	**Baseline**	**Device**	**Retransfusion**	**Baseline vs. Retransfusion**
Na+ (mEq/L)	142.5 ± 3.7	143.3 ± 4.6	137 ± 1.4	0.06
K+ (mEq/L)	4.1 ± 0.02	4.4 ± 0.6	5.8 ± 2.5	0.51
Cl- (mEq/L)	114 ± 9.6	115.5 ± 10.8	117 ± 7.1	0.70
Glucose (mmol/L)	150.8 ± 44.6	155.8 ± 51.7	187 ± 72.1	0.61
BUN (mmol/L)	5.3 ± 1.7	5.7 ± 3.1	4 ± 1.4	0.42
pH	7.39 ± 0.1	7.50 ± 0.1	7.31 ± 0.3	0.24
PO2	278.5 ± 118.1	183.5 ± 46	172 ± 72.1	0.41
PCO2	26.7 ± 8.8	13.9 ± 4.6	37.7 ± 16.6	0.52
Bicarbonate (mmol/L)	18.2 ± 0.1	16.9 ± 1.5	16.8 ± 1.7	0.46
Lactate (mmol/L)	2.7 ± 0.7	3.3 ± 1.8	4.1 ± 1.4	0.37
Base Excess	−8.9 ± 1.2	−12.3 ± 2.5	−6.39 ± 8.2	0.74
Hemoglobin (g/dL)	7.8 ± 2.4	8.2 ± 2.1	7.3 ± 1.8	0.74
Hematocrit (%)	23 ± 6.9	21.5 ± 7.7	21.3 ± 5.6	0.71
ACT (s)	268.5 ± 44.5	124 ± 93.3	193 ± 24.6	0.35

SD, Standard deviation; BUN, Blood urea nitrogen; O2, Oxygen; CO2, Carbon dioxide; ACT, Activated clotting time.

### Unintended hemorrhage

The device was kept on-shelf over three different IACUC-approved protocols (22, 23) to test this device in a real-world setting involving invasive procedures where the bleeding was a known risk but not necessarily anticipated. Ultimately, the device was used to resuscitate two male Swine (50–60 kg) when complications led to uncontrolled hemorrhage: one abdominal hemorrhage and one thoracic hemorrhage. The handheld autotransfusion device was promptly assembled and used to suction the blood from the region of bleeding and then pushed into a heparinized bag. The shed blood was then transfused into the animal using a standard pressure bag inflated to 150 mmHg. Due to the spontaneous nature of these events, laboratory tests were not obtained prior to or during device use. However, an ABG and Chem8 were taken post-transfusion for one of the two animals: pH of 7.3, lactate of 5.1 mmol/L, hemoglobin of 9.9 g/dL, and hematocrit of 29%. Both animals survived resuscitation, and experiments were completed.

### Device considerations

The Hemafuse pump has a capacity of 400 mL and can be used up to 25 times with autoclaving. The filter and accessories, such as the Yankauer suction tip, tubing, and stopcock, should be disposed of after every use and should not be reused if blood clots compromise the device, according to the Instructions For Use and package documentation. If the device is used in a continuous fashion and multiple empty blood bags are available, the device can collect all pooled blood in the surgical site. A single device is not limited by the volume of collection but instead is limited by the time of the surgery or a secondary hemorrhage. The device is designed for the initial resuscitation of a massive hemorrhage. In the event of a second severe hemorrhage, a second device can be used, or alternatively, the initial device can be flushed with saline or heparinized saline to remove any blood clots.

The Hemafuse device was intuitive to learn and easily assembled when needed. All laboratory personnel needed to be adequately trained on device use to avoid complications that may be preventable. The device can be assembled in < 5 min with a more efficient assembly time associated with experienced, trained personnel. Before device use, it was also critical that each team member was assigned a role, and individuals communicated which each other to suction the blood into the barrel, release the trapped air, and push the blood into the bag. This device can be used with a minimum of two individuals. Over the entire trajectory of this study, we timed the length of time it took from the start of suction to re-transfusion and recorded whether 2 or 4 members used the device. There were no differences between two and four team members, with an overall mean time to retransfusion of 128.7 ± 13.3 s. Overall, we experienced few problems with device operation in both controlled and unintended hemorrhage animals.

## Discussion

This report demonstrates the feasibility of the Hemafuse device for large animal resuscitation in a research laboratory for studies of controlled hemorrhage or unintended large-volume hemorrhage, with user and device considerations. This is important for recovering large animals following unintended bleeding to reduce costs and lower animal attrition. Following autotransfusion, we found no changes in markers of metabolic or hematologic derangement. After autotransfusion, the pH, lactate, hemoglobin, and hematocrit were all similar to baseline values. We found the device successfully collected and reinfused blood, with a 100% survival rate when used in animals with unintended hemorrhage in our real-world application. The device was user-friendly and required minimal training with few complications experienced in controlled and uncontrolled hemorrhage. The Hemafuse device can potentially serve as a low-cost emergency tool to store in the laboratory to resuscitate animals when complications ensue.

Unintended hemorrhage has a high mortality rate and requires prompt intervention to improve survival (10, 11). Resuscitation has historically been performed with crystalloid fluids in large volumes with the addition of red blood cell transfusions and/or pressors (24, 25). Over time, blood transfusions have been shown to be more effective as the primary resuscitative material (13). Specifically, early blood transfusions were found to be a successful resuscitation technique in the setting of non-compressible hemorrhage (9, 13). Many large animal research laboratories may not have access to a blood bank or other sufficient methods to resuscitate an animal if an unintended hemorrhage occurs. However, in an attempt to preserve precious resources in situations that may otherwise be deemed futile and lead to experiment termination, resuscitating a distressed animal to continue the experimental protocol can reduce the total number of animals required to meet the target sample size.

The financial stakes vary between animal research laboratories, with some, such as xenotransplantation laboratories, involving genetically engineered animals, each worth over $200,000 and potentially $20,000–$50,000 per organ (26, 27). When used for unintended hemorrhage, we successfully resuscitated two animals, which saved $2,600 (~$1,300 per animal and husbandry costs) and prevented additional animals from being used in the sample size. Alternatively, and likely more commonly, the ethical considerations, experimental costs, and laboratory maintenance costs are incentives to ensure successful experiments. Overall, this device has the potential to pay for itself when used to salvage research animals, especially in laboratories without substantial funding.

Because access to modern resuscitative technology is dependent on the level of laboratory funding, most researchers cannot afford an ICS machine or the recommended perfusionist for the machine unless there are high financial stakes where blood loss is likely, such as a xenotransplantation laboratory (17, 26–28). In addition to the costly upfront expensive of an ICS and the maintenance required (17, 18), the ICS machine carries the risk of hemolysis; however, this can be prevented by suctioning from a pooled source of blood (15, 16, 18). Similar to the ICS, recovering blood from surgical sponges is another proposed method of autotransfusion (29, 30). After processing the salvaged blood, studies found the red blood cell mass to be lower than suctioning, though the hematocrit was likely not physiologically significant (29, 30). A recent device has been introduced to further optimize the recovery and processing of blood from sponges, known as the ProCell device (31). While these methods of autotransfusion are likely not feasible for many researchers; it demonstrates the potential benefit of autologous blood transfusions for animals and pertinent outcomes to investigate (i.e., the hematologic effects).

In both the controlled and the unintended hemorrhage groups, the Hemafuse device was an effective method for resuscitation with little to no metabolic or hematologic alterations. As expected, the MAP and CVP significantly increased upon autologous blood transfusion. Although only studied in one animal, the iliac artery flow was 79% greater during resuscitation with blood transfusion compared to baseline data and normalized over time. Increased flow is associated with improved perfusion and can prevent hemorrhage-related complications, such as ischemic-reperfusion injury (4, 32).

Common complications of hemorrhage and transfusion therapy were assessed, such as acidosis, hypocalcemia, and hypo- or hyperkalemia (33). Upon resuscitation with autologous blood, there were no significant changes in pH or lactate, demonstrating the safety of the autologous blood and potential treatment of acidosis. Similarly, there were no significant electrolyte abnormalities following retransfusion. In our real-world application of this device, mortality was the most important outcome with an incidence of 0/2. While a small sample, this data shows no safety signal and demonstrates the feasibility of the device for recovering lost blood.

Furthermore, because machine-processing blood has been found to cause hemolysis and reduced red blood cell mass (15, 16, 29, 30), we closely monitored hemoglobin and hematocrit for hemolysis. Additionally, coagulopathy can be a consequential hemorrhage complication and was investigated. Compared to baseline, there were no significant differences in hemoglobin, hematocrit, or ACT following blood retransfusion. To verify the device does not significantly increase coagulopathy, further research into specific coagulation factors is required, such as measuring partial thromboplastin time (PTT), prothrombin time (PT), or thromboelastography (TEG) testing. The Hemafuse device was both efficacious and safe to use, with minimal changes in the physiologic profile of the animals when compared to baseline data.

When used for intraperitoneal pelvic hemorrhage, the Hemafuse device was a straightforward, cost-effective method of resuscitation. In our experience, we found that minimal training was needed, and only two team members were required to use the device successfully. Assigning team members roles in the resuscitative protocol was an effective way to increase the time to retransfusion and decrease complications. On average, the time to retransfusion of autologous blood was approximately 2 min from when suction was initiated and up to 5–7 min when accounting for the time of device assembly. This model of re-transfusion following free bleeding may be useful in constructed animal experiments for studying shock and hemorrhage with retransfusion.

Optimal suction was achieved when suctioning blood pooled in the pelvis. Otherwise, air accumulated in the device, which increased the required device cycles (i.e., suctioning blood and pushing blood into the bag). Additionally, when there was minimal blood pooling in the pelvic cavity, the suction tip was more likely to be obstructed by tissue (e.g., peritoneum). The excess air was not a significant issue when the device was spontaneously used in the thoracic cavity in one animal, likely due to anatomical differences. Pre-heparinizing the blood bag was crucial to ensure device success, as large blood clots formed quickly when the blood bag was not heparinized. In animals that were not heparinized, there was a small chance of clot formation within the filter or barrel; however, this can be mitigated by a deeper pool of blood to move the blood quickly into the heparinized bag. When the protocol was optimized, the device effectively resuscitated large research animals during controlled and uncontrolled hemorrhage.

The Hemafuse device was designed for human use; more specifically, it was created to manage peri- or post-partum hemorrhage in low-resource settings (19, 34). Early clinical trials have shown the device's efficacy with no alterations in hemoglobin or hematocrit (34, 35). The device received regulatory approval in Ghana and Kenya in 2017 with ongoing expansion efforts (19). Because this device is already being used successfully in humans, the threshold for application in research animals should be low. Our primary consideration was related to whether swine blood, which is higher in coagulation factors (36), would clot within the device. We did not experience any clotting within the device when it was used per protocol. Additionally, there were no differences in ACT when baseline, device, and post-transfusion phases were compared. Overall, our data supports the adaption of the device from humans to swine for research.

As researchers, the ethical considerations of animal research should be the primary motive for limiting the number of animals used for experimental protocols. There are three primary ethical considerations, known as the principle of the 3 Rs, for animal research: replacement, reduction, and refinement (37). When an alternative research method cannot be used (i.e., replacement), then it is imperative that researchers strive to reduce the number of animals used in experimental protocols. In experiments with a high risk of hemorrhage, such as surgical protocols, having a plan to resuscitate animals to ensure a successful experiment would be considered more ethical than enrolling additional animals in the study. As previously mentioned, resuscitating a research animal could disrupt physiology in a setting where normal physiology is crucial to measuring any effects the experiment has on physiology. If excessive fluid resuscitation or pressors are used, there are several consequences (e.g., disturbance of chemistry and acid-base, coagulopathy, vasospasms, etc.) that could lead to unused data from the experiment. However, it is important to refine experiments by decreasing inhumane procedures and increasing animal enrichment (37). If resuscitating an animal could cause more harm, then it should not be performed. Whether driven by financial or ethical considerations, the Hemafuse device has the potential to preserve animal lives when unexpected events occur during experimental protocols.

This study is not without limitations. First, the Hemafuse device itself has a few limitations. The device is designed for the initial hemorrhage and a second device must be used if blood clots compromise the filter or tubing. Second, how the filter functions in the setting of bacterial contamination is unknown and requires further study. Second, the 170-micron filter prevents larger particles from entering the blood bag, but does not filter bacterial contamination from the blood, and the collected blood is not recommended for infusion in the event of bacterial contamination. A further study could evaluate the bacterial contamination in the blood after filtration. Third, unlike automatic suctioning, the device may not provide adequate visualization of the field due to the manual suction. If the field needs to be cleared at a faster rate, automatic suction can be used, followed by the use of the Hemafuse device to quickly suction blood from the reservoir to transfer the blood into blood bags to autotransfusion.

In regard to the limitations of our study design, the data collected when the Hemafuse device was used for unintended hemorrhage was not standardized. While the device was left on the shelf for emergent real-world use, the primary goal during this time was to resuscitate the animal and continue the experimental protocol. Thus, data collection during autotransfusion and post-transfusion was not the main priority at the time. Because of the irregularity in this data, we chose to develop an appropriate model of pelvic hemorrhage to conduct a more rigorous experiment. While the model development and subsequent data collection were standardized, the sample size was small and increased the risk of a type two error. Similarly, there were data, such as the iliac artery blood flow, that was only studied in one animal and could not undergo statistical analysis. Lastly, we did not have a control group (e.g., a cohort who received only crystalloids) and cannot conclude that the Hemafuse device should be used over fluid resuscitation. However, based on literature and current resuscitation guidelines, blood transfusions for hemorrhage have improved outcomes over fluid resuscitation (13, 24, 38). While these limitations exist, the study shows the device is a low-risk option for resuscitating animals in dire situations.

## Conclusion

The Hemafuse device is an efficacious, low-cost option for large animal resuscitation of shed blood in a research laboratory. Whether hemorrhage is controlled or unintended, this device is low-risk and intuitive for resuscitation using autologous blood. While the sample size was small, we found no differences in metabolic or hematologic laboratory values at baseline compared to post-transfusion in both the real-world and controlled hemorrhage models. Further research on the full effects of this device for large animal resuscitation is needed. However, the Hemafuse device can potentially preserve animal lives with minimal risk in devastating situations, and it is recommended that research laboratories stock the device for emergency use.

## Data availability statement

The raw data supporting the conclusions of this article will be made available by the authors, without undue reservation.

## Ethics statement

The animal study was reviewed and approved by University of Maryland, School of Medicine, Baltimore, MD, USA.

## Author contributions

DS and JM conceived the study and designed the experiments. RT and DS conducted experiments. RT analyzed the data and wrote the manuscript. All authors approved of and participated in the critical revision of the manuscript.
